# Serums, Vaccines, and Toxins in Treatment and Diagnosis

**Published:** 1904-10

**Authors:** 


SERUMS, VACCINES, AND TOXINS IN TREATMENT AND DIAGNOSIS. By Wm. Cecil Bosanquet, M. A., M. D., Oxon.; F. R. C. P., Lond.; Physician to Out-Patients, Victoria Hospital for Children, London. Limp cloth, $2.00 net. W. T. Keener & Co., 90 Wabash Avenue, Chicago.

This neat and very handy little book in flexible cover will post the doctor on the modern methods of treatment by the remedies therein discussed. Very few of the older men are thoroughly "up" in serum therapy, and all can learn much that is valuable and timely from this work. It brings the treatment up *pari passu* with the progress made in bacteriology and the nature and mode of work of infective agents. It also treats of "immunity." I can recommend it to my readers as a desirable acquisition and daily help. D.

